# Implementation research: a protocol for two three-arm pragmatic randomised controlled trials on continuous glucose monitoring devices in people with type 1 diabetes in South Africa and Kenya

**DOI:** 10.1186/s13063-024-08132-7

**Published:** 2024-05-21

**Authors:** Elena Marbán-Castro, Lorrein Muhwava, Yvonne Kamau, Elvis Safary, Paul Rheeder, Maria Karsas, Tanja Kemp, Johanè Freitas, Michelle Carrihill, Joel Dave, Daniel Katambo, Joan Kimetto, Razana Allie, Kenya ACCEDE study group, Nancy Ngugi, Gaman Muhammed, Eva Njenga, Catherine Karekezi, Nancy Kunyiha, Newton Ngugi, Elizabeth Onyango, South Africa ACCEDE study group, Amanda Mashego, Kirsten De Klerk, Salih Hendriks, Newton Ngugi, Bilqees Sayed, Joseph Ndungu, Ntombi Sigwebela, Dorcas Akach, Sarah Girdwood, Berra Erkosar, Brooke E. Nichols, Cathy Haldane, Beatrice Vetter, Sonjelle Shilton

**Affiliations:** 1 FIND, Campus Biotech, Chemin Des Mines 9, 1202 Geneva, Switzerland; 2 University of Pretoria Diabetes Research Centre, Gezina, Pretoria, South Africa; 3 Red Cross Childrens Hospital, Paediatric Clinic, Cape Town, South Africa; 4 Division of Endocrinology, Groote Schuur Hospital and the University of Cape Town, Cape Town, South Africa; 5 Kenya Diabetes Management and Information Centre, Nairobi, Kenya

**Keywords:** (3–10): glucose monitoring, CGM, Continuous glucose monitoring, Protocol, Standard of care, Type 1 diabetes, Randomised controlled trial

## Abstract

**Background:**

Self-monitoring of glucose is an essential component of type 1 diabetes (T1D) management. In recent years, continuous glucose monitoring (CGM) has provided an alternative to daily fingerstick testing for the optimisation of insulin dosing and general glucose management in people with T1D. While studies have been conducted to evaluate the impact of CGM on clinical outcomes in the US, Europe and Australia, there are limited data available for low- and middle-income countries (LMICs) and further empirical evidence is needed to inform policy decision around their use in these countries.

**Methods:**

This trial was designed as a pragmatic, parallel-group, open-label, multicentre, three-arm, randomised (1:1:1) controlled trial of continuous or periodic CGM device use versus standard of care in people with T1D in South Africa and Kenya. The primary objective of this trial will be to assess the impact of continuous or periodic CGM device use on glycaemic control as measured by change from baseline glycosylated haemoglobin (HbA1c). Additional assessments will include clinical outcomes (glucose variation, time in/below/above range), safety (adverse events, hospitalisations), quality of life (EQ-5D, T1D distress score, Glucose Monitoring Satisfaction Survey for T1D), and health economic measures (incremental cost-effectiveness ratios, quality adjusted life years).

**Discussion:**

This trial aims to address the substantial evidence gap on the impact of CGM device use on clinical outcomes in LMICs, specifically South Africa and Kenya. The trial results will provide evidence to inform policy and treatment decisions in these countries.

**Trial registration:**

NCT05944731 (Kenya), July 6, 2023; NCT05944718 (South Africa), July 13, 2023.

## Administrative information

**Table:** 

**Title {1}**	**Implementation research: A protocol for two three-arm pragmatic randomised controlled trials on continuous glucose monitoring devices in people with type 1 diabetes in South Africa and Kenya**
Trial registration {2a and 2b}	Name of the registry: Clinicaltrials.gov Trial registration number: NCT05944718 (South Africa) Date of registration: July 13, 2023 URL of trial registry record: https://clinicaltrials.gov/study/NCT05944718?cond=Diabetes%20Mellitus%20Type%201&term=south%20africa&rank=1 Name of the registry: Clinicaltrials.gov Trial registration number: NCT05944731 (Kenya) Date of registration: July 6, 2023 URL of trial registry record: https://clinicaltrials.gov/study/NCT05944731?cond=Diabetes%20Mellitus%20Type%201&term=Kenya&rank=1
Protocol version {3}	Version 4.0 (4 September 2023; Kenya); version 1.1 (16 July 2023; Pretoria, South Africa); version 1.2 (28 September 2023; Cape Town, South Africa)
Funding {4}	FIND
Author details {5a}	Elena Marbán Castro1, Lorrein Muhwava1, Yvonne Kamau1, Elvis Safary 1, Paul Rheeder2, Maria Karsas2, Tanja Kemp2, Johanè Freitas2, Michelle Carrihill3, Joel Dave4, Daniel Katambo5, Joan Kimetto5, Kenya ACCEDE trial group*, South Africa ACCEDE trial group**, Joseph Ndungu1, Ntombi Sigwebela1, Dorcas Akach 1, Sarah Girdwood1, Berra Erkosar1, Brooke E Nichols1, Cathy Haldane1, Beatrice Vetter1, Sonjelle Shilton1 1FIND, Geneva, Switzerland; 2University of Pretoria Diabetes Research Centre, Gezina, Pretoria, South Africa; 3Red Cross Childrens Hospital, Paediatric Clinic, Cape Town, South Africa; 4Division of Endocrinology, Groote Schuur Hospital and the University of Cape Town, Cape Town, South Africa; 5Kenya Diabetes Management and Information Centre, Nairobi, Kenya
Name and contact information for the trial sponsor {5b}	FIND Beatrice Vetter Beatrice.Vetter@finddx.org Sonjelle Shilton Sonjelle.Shilton@finddx.org Campus Biotech, Chemin des Mines 9 1202 Geneva, Switzerland
Role of sponsor {5c}	Beyond usual governance activities and the involvement of the authors affiliated with the sponsor, the sponsor (FIND) was involved in the trial design, writing of the report, and the final decision to submit the report for publication

## Introduction

### Background and rationale {6a}

Self-monitoring of glucose via regular daily fingerstick glucose testing is an essential component of managing people with type 1 diabetes (T1D) [1]. However, in recent years the development of continuous glucose monitoring (CGM) devices has provided an alternative option for optimising insulin dosing and glycaemic control in people with T1D [2]. A systematic review of the use of real-time CGM and intermittent CGM showed that CGM was associated with a reduction in glycated haemoglobin (HbA1c; weighted mean difference: -0.17% [95% confidence interval {CI} -0.29, -0.06]), and an increase in the time users spent with daily glucose levels ‘in-range’ (usually 70–180 mg/dL; weighted mean difference: + 70.7 min [95% CI +46.7, +94.8]) [1]. Of the two CGM schedules assessed, real-time CGM use was shown to provide the greatest reduction in HbA1c (weighted mean difference: -0.23% [95% CI -0.36, -0.10]) and the greatest increase to the time ‘in-range’ (weighted mean difference: +83.5 min [95% CI +52.7, +114.3]) [1]. These findings have also been supported by the results of several individual studies, where CGM was found to improve glycaemic control in people with T1D and reduce the frequency of hypoglycaemic events [2–4].

However, this body of evidence consists primarily of studies conducted in the United States (US) [1, 3, 4], Europe [1, 2], or Australia [1], and there are limited data on the effects of CGM on clinical outcomes in low- and middle-income countries (LMICs), where CGM devices may not be readily available. A recent scoping review of CGM in LMICs did not identify any randomised trial conducted in Sub-Saharan Africa [5], and to our knowledge only one study on the use of CGM in people with T1D has been conducted in the region, which was a feasibility study in Kenya and Uganda and not a randomised control trial [6]. Similarly, we have identified no randomised trials conducted in South Africa that have evaluated the impact of CGM compared with standard of care glucose monitoring on HbA1c (and other clinical outcomes) in people with T1D, with the majority of the evidence base consisting of qualitative, mixed methods, and perception studies [7–10]. Overall, there exists a substantial evidence gap on the use of CGM in people with T1D in LMICs, particularly those in Sub-Saharan and Southern Africa, and while some devices have received US Food and Drug Administration (FDA) approvals or European Commission “Conformité Européenne” CE marks [11], further empiric evidence is needed to inform policy decision around their use in these countries as evidence generated in higher resource settings may not be applicable [11–13].

The rationale for selecting Kenya and South Africa as study locations is rooted in different reasons. In South Africa, 31,500 people live with T1D, with an annual growth rate of 6.1% [14]. In Kenya, there are 23,100 people living with T1D, with an annual growth rate of 4.2% [15]. There are different levels of care provision for T1D in both countries, which translates into different approaches to T1D management. Additionally, there are different levels of CGM availability in both countries.

Here we present the protocol for a pragmatic trial that aims to assess the impact of continuous or periodic CGM device use on glycaemic control (and other clinical, quality of life, and health economic outcomes) in people with T1D in South Africa and Kenya. The trial was co-designed by diabetes stakeholders in South Africa and Kenya and are sponsored by FIND. The trial was designed to incorporate CGM use within the routine care in diabetes clinics in multiple centres, necessitating slight variations in design for each centre as routine care differed. The trial has been designed in accordance with the principles of the Declaration of Helsinki and country-specific regulatory and ethics requirements and has been approved by all relevant Institutional Review Boards (IRBs) and Independent Ethics Committees (IECs).

### Objectives {7}

The overall objective of this trial is to assess the impact of CGM (Intervention) on glucose levels (Outcome) compared with standard of care (Comparator) in people with T1D in South Africa and Kenya (Population).

#### Objectives

The primary objective is to evaluate the impact of continuous and periodic CGM on changes to HbA1c levels in people with T1D in South Africa and Kenya, compared with standard of care.

Secondary objectives are as follows (versus standard of care where appropriate):

- To evaluate the impact of continuous or periodic CGM device use on glucose variability.
- To evaluate the impact of continuous or periodic CGM device use on the number of unplanned visits to outpatient clinics and/or hospitals as a result of diabetes complications.
- To evaluate the acceptability and feasibility of continuous or periodic CGM device use from the perspective of the participant, caregiver, and healthcare provider.
- To evaluate the cost of continuous or periodic CGM device use from a user and provider perspective.

### Trial design {8}

This trial is a pragmatic, parallel-group, open-label, multicentre, three-arm, superiority randomised (1:1:1) controlled trials of continuous or periodic CGM device use (the Abbott Freestyle Libre 1 device) versus standard of care in people with T1D in Kenya and South Africa. The primary endpoint is the mean change in HbA1c levels. The trial was designed using the PRECIS-2 tool, a nine-domain tool intended to be used in the trial design phase, which is intended to align design decisions with applicability of trial results [16]. 

The trial is expected to take approximately 18 months to complete (September 2023 to April 2025 in South Africa, and February 2024 to July 2025 in Kenya), including trial set-up, recruitment, and follow-up for all participants.

## Methods: Participants, interventions, and outcomes

### Study setting {9}

The trial in Kenya will be conducted at three public sector clinics that are part of the ‘Changing Diabetes in Children’ program: the Kenya Diabetes Management and Information Centre, and the Machakos and Kiambu level five referral hospitals. This program was launched in 2012 to improve T1D care in children and young adults (under the age of 18), by working with the Kenyan Ministry of Health to build capacity and strengthen the local health system through the provision of medical equipment, point-of-care glucose meters, healthcare training, and dissemination of best practice methods [17].

The trial in South Africa will be conducted at three public sector clinics: the diabetes clinic at Groote Schuur Hospital in Cape Town; the paediatric diabetes clinic at Red Cross War Memorial Children’s Hospital in Cape Town; and the adult and paediatric diabetes clinic at Steve Biko Academic Hospital in Pretoria.

### Eligibility criteria {10}

#### Inclusion criteria

Participants meeting all of the following criteria will be eligible for enrolment in the trials:

- People with T1D attending one of the clinics for regular diabetes care, with a current (within the past 3 months in South Africa and 18 months in Kenya) HbA1c level of ≥10%/86 mmol/mol and no record of an HbA1c < 8%/ <64 mmol/mol within the past 9 months.
  o Caregivers of children or adolescents with T1D will be eligible for inclusion in the trial (for acceptability and feasibility assessments) only if the child or adolescent is also enrolled in the trial.
  o Healthcare providers will be eligible for inclusion in the trial (for acceptability and feasibility assessments) only if they are engaged in trial-related diabetes care at one of the trial clinics (trial Investigators are excluded).

#### Exclusion criteria

Participants will be excluded from the trial if they meet any of the following criteria:

- People with T1D under 4 years old, as per the trial CGM device (Abbott Freestyle Libre) manufacturer’s instructions for minimum age of use.
- People diagnosed with T1D within the past 2 years.
- People who have used a CGM device within 18 months (in Kenya) and 6 months (in South Africa) of trial enrolment.
- People who anticipate they would have access to a CGM device through means outside of the trial, during the duration of the trial (15 months).
- People with type 2 diabetes.
- People with known pregnancy at the time of trial enrolment.
- People who are not willing to agree to the CGM device (Abbott Freestyle Libre) terms and conditions.

### Who will take informed consent? {26a}

The trial Investigator or his/her representative will explain the nature of the trial to all participants who meet the inclusion criteria (or their legally authorised representative) and answer all questions they may have about the trial. After being informed that their participation is voluntary and being given ample time to decide whether they wish to take part in the trial, any participants (or their legally authorised representative) who do wish to enrol will be required to sign and date a statement of informed consent that meets the requirements of local regulations and relevant IRB/IECs. For children and adolescents aged 7–17 years (inclusive), they will also be required to sign and date a statement of informed assent that meets the requirements of local regulations and the relevant IRB/IEC. All written informed consent and informed assent must be obtained before the participant is enrolled and any trial-related procedures are performed.

### Additional consent provisions for collection and use of participant data and biological specimens {26b}

The informed consent form includes a statement that all information obtained during the course of the trial will be regarded as confidential, that only the site trial team will be able to identify participants, that results will be published or presented in such a fashion that participants remain unidentifiable, and that hard copies of all participant records will be kept for 10 years in a locked facility at the trial site.

Consent for biological specimens is not applicable as no samples collected for HbA1c measurement will be stored.

## Interventions

### Explanation for choice of comparators {6b}

The comparator is standard of care diabetes management at each trial clinic (glucose monitoring) by participants assigned to Arm 3, their HbA1c levels and all the data collected from their case report forms (CRFs).

### Intervention description {11a}

The intervention is a CGM device (Abbot Freestyle Libre), which is considered low risk to trial participants as it is approved by the FDA, has the CE mark and is prescribed in both countries by private healthcare providers for the management of diabetes. It consists of a small interstitial fluid glucose sensor that is applied on the back of the upper arm, and a hand-held reader that collects glucose data when placed within 1.5 inches (4 cm) of the sensor. In the continuous CGM trial arm, participants will wear the CGM device all the time for the 9 months (replacing it every 2 weeks, concurrent with senor wear time). In the periodic CGM trial arm, participants will wear the CGM device for 2 weeks, scheduled to start 1 week before each clinic visit (except for the enrolment visit), and for 1 week after each clinic visit (except for the final follow-up visit).

### Criteria for discontinuing or modifying allocated interventions {11b}

A participant may discontinue CGM device use and be withdrawn for safety or administrative reasons at any time at the discretion of the Investigator. A participant may also decide to withdraw from the trial at any time. If a participant becomes pregnant, they will be permitted to remain in the trial to ensure external validity of the data.

### Strategies to improve adherence to interventions {11c}

An education session on the use of the CGM device will be provided for each participant during the enrolment visit (Day 1), which will be conducted by a qualified instructor and include guidance on how to self-apply the device, how to correctly interpret the information collected by the device, diabetes health and management, and awareness and management of potential adverse events. In addition, all clinicians (including registrars, nurse educators and research assistants) involved in the trial will receive an education package on clinical management and use of the CGM device.

### Relevant concomitant care permitted or prohibited during the trial {11d}

As this is a pragmatic trial embedded in routine clinical care, participants will be permitted to receive any concomitant care as clinically required. Potential confounding treatments will be assessed in a ‘Medical events and medical resources’ survey and include acetaminophen, ascorbic acid, intravenous vitamin C, sulphonamide antibiotics, and oxygen therapy, among others.

### Provisions for post-trial care {30}

As standard of care diabetes treatment is permitted during the studies, this will be continued once the trial has been completed. Any decision on whether to use a CGM device after the trial sits entirely with the participant and their attending clinician.

### Outcomes {12}

#### Primary outcome

The primary endpoint will be the mean change from baseline (pre-CGM device use) HbA1c at various timepoints up to 15 months (percentage and mmol/mol). This will be assessed in all trial arms and presented as absolute values and percentage changes, together with summary statistics and statistical comparisons (between group).

#### Secondary outcomes

Unless otherwise stated, the secondary outcomes listed below will be presented using descriptive statistics (i.e., means, standard deviations) or as intervention effects with 95% CI.

- CGM device outputs: (i) Coefficient of variation (%CV) for glucose levels; (ii–iv) the time spent ‘in-range’, below-range’, and ‘above range’ (percentage per day and absolute values [hours/minutes]).
- Number of hospitalisations due to diabetes complications.
- Health-related quality of life (HRQoL): (i) mean change from baseline in EQ-5D-Y/EQ-5D/EQ-5D-interviewer-administration scores [18–20]; (ii) mean change from baseline in type 1 diabetes distress (T1D-DDS) scores for participant and parent (where possible) [21]; (iii) qualitative assessment using focus group discussions with participants and caregivers.
- Adherence to CGM device use: the proportion of scheduled time registered using the CGM device (automatic device output).
- Acceptability: (i) mean change from baseline in Glucose Monitoring Satisfaction Survey for T1D (GMSS-T1D) score; [22] (ii) qualitative assessment using semi-structured interviews with healthcare providers and focus group discussions with participants.
- Cost: surveys will capture both direct and indirect costs of diabetes care at follow-up Visits 3 to 6 for trial participants, their caregivers (if possible), and healthcare providers. Incremental cost-effectiveness ratios (ICERs) and quality adjusted life years (QALYs) will be used for between-group comparisons.

#### Exploratory outcomes

In Kenya, the accuracy of the HbA1c point-of-care device used at each participating clinic will be assessed using a laboratory-based HbA1c method as reference. Mean relative bias, 95% limits of agreement and regression estimates will be presented. In South Africa, there are no exploratory outcomes, HbA1c test results analysed for the trial will be the ones used as per routine care.

#### Safety outcomes

Safety will be assessed by the recording of adverse events (AEs) and serious adverse events (SAEs) occurring during the trial period, as defined by the Common Terminology Criteria for Adverse Events (CTCAE). Given the pragmatic nature of the studies, reporting will be limited to SAEs. All events of grade 3 or above will be considered an SAE.

### Participant timelines {13}

**Fig. 1:**
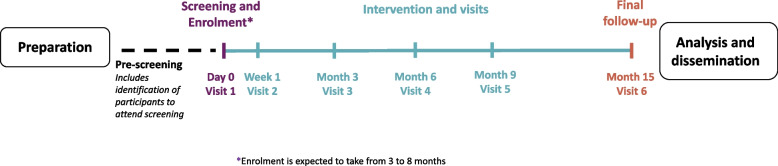
Study timeline schematic. *Enrolment is expected to take from 3 to 8 months. Q, quarter

The duration for each participant will be 15 months, consisting of 9 months of intervention followed by 6 months’ follow-up. There will be six scheduled trial visits in total, at Day 1, Visit 2 (Week 1), and at 3 (Week 11/12), 6 (Week 23/24), 9 (Week 35/36), and 15 months (Week 57/58); Fig. 1).

### Sample size {14}

Assuming a change in HbA1c levels of 2% after continuous use of a CGM device (determined by expert consultation) and an effect size of 0.2, a sample size of 246 participants (82 in each arm) would be required to achieve 80% power to detect within- and between-group differences at the 5% significance level (using repeated measures analysis of variance [ANOVA]).

For qualitative assessments, sample sizes will be pragmatically derived and will vary based on the number of available respondents and attainment of information/thematic saturation. In Kenya, the target populations will be 30 for focus group discussions and HRQoL surveys (10 in each arm) and 10 for semi-structured healthcare provider interviews. In South Africa, the target populations will be 45 for focus group discussions and HRQoL surveys (15 in each arm) and 10 for semi-structured healthcare provider interviews.

### Recruitment {15}

Recruitment will be staggered over a period of 4 weeks or until the target sample size has been reached. Participants will be recruited using purposive sampling at each of the sites, based on eligibility criteria. To raise awareness of the studies, recruitment flyers, which will contain basic trial information (purpose, inclusion criteria), as well as the contact details for the Principal Investigator and/or trial staff will be distributed to potential participants. Trial staff will contact eligible participants via phone and invite them to an information session at the clinic, or consecutively explain study procedures at their routine clinic visit. At that visit eligibility screening and informed consent/assent will take place. Reasons for refusal to participate will be recorded in the screening and enrolment logs and reviewed regularly by trial staff, so that strategies to maximise recruitment can be developed (if required).

The caregiver of every child or adolescent enrolled will be invited to participate in the HRQoL surveys and will be considered for inclusion in the focus group discussions. Parents of children under 8 years of age will also be asked to complete the surveys. All healthcare providers involved in the trial will be invited in person or via phone/email to take part in the semi-structured acceptability discussions.

## Assignment of interventions: allocation

### Sequence generation {16a}

Upon enrolment, each participant will be assigned a unique randomisation number in ascending numerical order, which determines their assignment to one of the three arms (1:1:1) according to the randomisation schedules generated prior to the trial by the FIND trial statistician. As this is an open-label trial, in order to minimise the risk of bias research assistants will be required to log into an electronic database (OpenClinica), where they will enter the participant’s unique randomisation number and the system will then inform them of the participant’s arm.

### Concealment mechanism {16b}

Only the trial statistician who generated the allocation sequences will have access to the allocation sequences.

### Implementation {16c}

As detailed in ‘Sequence generation {16a}’ section, the allocation sequences will be generated prior to trial initiation by the FIND trial statisticians. Trial Investigators/staff will enrol participants into the trial and assign them their randomisation numbers, which will determine their trial group based on the allocation sequences.

## Assignment of interventions: blinding

### Who will be blinded {17a}

As pragmatic, open-label trial, no blinding will be required.

### Procedure for unblinding if needed {17b}

As this will be open-label trial, unblinding will not be required.

## Data collection and management

### Plans for assessment and collection of outcomes {18a}

Trial outcomes are defined in the ‘Outcomes {12}’ section. All outcomes data relating to glucose levels will be extracted directly from the CGM device, as will data on adherence to CGM device use. However, in order to minimise bias in the intervention effect assessment, source data verification (from direct laboratory reports) will be implemented for the primary outcome measure (HbA1c). Other assessments will take place at the trial visits and timepoints outline in Table 1 and Table 2. Focus group discussions (~ 1 h) and semi-structured interviews (~ 1 h) will be conducted by trained staff at the Month 9 visit.

**Table 1:** Schedule of assessments (Kenya study; NCT05944731)

	**STUDY PERIOD**						
	**Before enrolment**	**Enrolment / allocation**	**Post allocation study visits**				**Final follow-up**
	Day 0	Day 1 (Visit 1)	Week 1 (Visit 2)	Month 3 (Visit 3)	Month 6 (Visit 4)	Month 9 (Visit 5)	Month 15 (Visit 6)
**ENROLMENT**							
Pre-screening^a^	All						
Screening		All					
Enrolment		All					
Informed consent		All					
Baseline demographics		All					
Education session		All	All	All	All	All	All
**INTERVENTION**							
CGM device application or distribution^b^		Arms 1 and 2	Arm 2	Arm 2	Arm 2	Arm 2	
**ASSESSMENTS**							
Doctors visit (medical chart review)			All	All	All	All	All
HbA1c assessment		All		All	All	All	All
Medical events and resources survey				All	All	All	All
GMSS		All		All	All	All	All
HRQoL assessments		All		All	All	All	All
DDS		All		All	All	All	All
Acceptability questionnaire				Arms 1 and 2	Arms 1 and 2	Arms 1 and 2	
Focus group discussion^c^						All	

*CGM* Continuous glucose monitoring, *DDS* Diabetes distress score, *GMSS* Glucose Monitoring Satisfaction Survey, *HRQoL* Health Related Quality of Life

^a^Includes identification of potential participants to be invited to the screening and enrolment day

^b^In the continuous CGM arm, the device is applied once on Day 1 and used until Month 9, for the periodic arm, a new device is provided at the visits shown and used for 1 week before each clinic visit (except for the enrolment visit), and for 1 week after each clinic visit (except for the final follow-up visit)

^c^Performed in a subset of participants

**Table 2:** Schedule of assessments (South Africa study; NCT05944718)

	**STUDY PERIOD**						
	**Before enrolment**	**Enrolment / allocation**	**Post allocation study visits**				**Final follow-up**
	Day 0	Day 1 (Visit 1)	Week 1 (Visit 2)	Month 3 (Visit 3)	Month 6 (Visit 4)	Month 9 (Visit 5)	Month 15 (Visit 6)
**ENROLMENT**							
Pre-screening^a^	All						
Screening		All					
Enrolment		All					
Informed consent		All					
Baseline demographics		All					
Education session		All	All	All	All	All	All
**INTERVENTION**							
CGM device application or distribution^b^		Arms 1 and 2	Arm 2	Arm 2	Arm 2	Arm 2	
**ASSESSMENTS**							
Doctors visit (medical chart review)			All	All	All	All	All
HbA1c assessment			All	All	All	All	All
Medical events and resources survey				All	All	All	All
GMSS		All			All		All
HRQoL assessments		All				All	All
DDS		All			All	All	All
Acceptability questionnaire					Arms 1 and 2		Arms 1 and 2
Focus group discussion^c^						All	

*CGM* Continuous glucose monitoring, *DDS* Diabetes distress score, *GMSS* Glucose Monitoring Satisfaction Survey, *HRQoL* Health Related Quality of Life

^a^Includes identification of potential participants to be invited to the screening and enrolment day

^b^In the continuous CGM arm, the device is applied on on Day 1 and used until Month 9, for the periodic arm, a new device is provided at the visits shown and used for 1 week before each clinic visit (except for the enrolment visit), and for 1 week after each clinic visit (except for the final follow-up visit)

^c^Performed in a subset of participants

### Plans to promote participant retention and complete follow-up {18b}

To minimise participant drop out (and also improve adherence to CGM device use, as new CGMs will be provided at each trial visit), participants will be enrolled in the trial who are already receiving T1D care at the participating clinics, and reminders will be sent to participants before the scheduled visits. In addition, travel reimbursement will be provided for each trial visit to minimise the chance that socio-economic issues may influence participant dropout and adherence to CGM device use. Should a participant miss a trial visit, the sites will attempt to contact them and reschedule the missed visit as soon as possible, as well as counsel the participant on the importance of maintaining the assigned visit and CGM device schedule.

### Data management {19}

The trial sponsor (FIND) will be responsible for the data management, including risk-based monitoring, quality control checks, data cleaning, and protocol compliance. Clinical data and laboratory results will be captured by site staff onto paper-based CRFs during trial visits and entered onto an electronic case report form designed by FIND in the electronic data capture (EDC) system. Site staff will undergo a training session provided by FIND on the use of the EDC system, and will be responsible for entering their data from a paper CRF into the EDC system whenever direct capture into EDC is not possible. The trial Investigators will be responsible for verifying that data entries are accurate and correct, and that data entered in EDC are consistent with the source documents (or the discrepancies adequately explained). Records and documents, including signed informed consent forms, pertaining to the conduct of the trial will be retained by the Investigators for the time mandated by local regulations and institutional policies, and no records may be transferred to another location or party without written permission from FIND.

### Confidentiality {27}

All information obtained will be regarded as strictly confidential and handled and stored according to all relevant Data Protection laws. Only the trial site staff directly involved in the conduct of the trial will be able to identify participants, and any data will be transferred for analysis or published in such a way that participants cannot be identified. Hard copies of all participant records will be kept for 10 years in a locked facility at all sites.

### Plans for collection, laboratory evaluation and storage of biological specimens for genetic or molecular analysis in the trial/future use {33}

No biological specimens will be collected, unless they are collected as part of standard of care at each participating diabetes clinic.

## Statistical methods

### Statistical methods for primary and secondary outcomes {20a}

A separate Statistical Analysis Plan will provide a detailed description of the planned statistical analyses and only a brief outline will be presented here. Please see the ‘Outcomes {12}’ section for definitions of the primary, secondary and exploratory outcomes and for further details on data presentation.

For the purposes of this trial, the following populations will be defined:

- Enrolled population: all participants enrolled in the trial who completed informed consent.
- Assigned population: all participants who have been assigned to a trial arm and completed all Day 1 trial visit procedures.
- Fully Complied: all participants who fully complied with the protocol.
- Partly Complied: all participants who recorded HbA1c values at Visit 1 and 5 (at least).
- Intervention: all participants in the CGM device arms who fully complied with the protocol.
- Safety: all participants who have been assigned to a trial arm.

#### Primary outcome

Between-group analyses of the primary outcome will be conducted using the Fully Complied or Partly Complied populations and two-way repeated-measures ANOVA with time and arm as independent variables.

#### Secondary outcomes

Glucose variation will be assessed using the Intervention population, with %CV calculated as the ratio of the standard deviation to the mean. HRQoL outcomes will be analysed using the Fully Complied or Partly Complied populations and two-way repeated-measures ANOVA with time and arm as independent variables. Within-group mean GMSS scores will be analysed using t-tests and between-group scores using repeated-measures two-way or one-way ANOVA (depending on the availability of data) with time and arm as independent variables (all using the Fully Complied or Partly Complied populations).

All other secondary outcomes will be analysed using descriptive statistics: hospitalisations using the Partly Complied population, adherence using the Intervention population, and cost surveys using the Fully Complied or Partly Complied population.

For the cost analysis, standard bottom-up costing methods from a partial societal perspective will be used to estimate the costs of both direct and indirect resources utilised by participants over the trial period. Unit costs will be assigned to these resource outputs using values sourced from public sources: laboratory test costs from the relevant national laboratory service, staffing costs from the Salaries and Renumeration Commission, medicine and supply costs from Kenya Medical Supplies Authority (KEMSA)/Mission for Essential Drugs (MEDs), minimum wage data from the relevant government agency, and the cost of procedures and in-patient hospital stays from the National Health Insurance Fund. Where necessary, unit costs will be sourced from other publicly available resources and the literature. ICERs will be calculated using the primary outcome measure and the time ‘in-range’, and QALYs will be calculated from the HRQoL outcomes.

#### Exploratory outcomes

In Kenya, the HbA1c point-of-care device accuracy analysis will be conducted using Bland–Altman plots (mean relative bias), 1.96*standard deviations (limits of agreement), and Passing Bablok regression.

#### Qualitative analysis

Focus group discussions and semi-structured interviews will be recorded, transcribed and coded. Following this, a thematic analysis will be completed on the responses using inductive and deductive coding.

### Interim analyses {21b}

No interim analyses of primary, secondary or safety outcomes will be performed.

### Methods for additional analyses (e.g. subgroup analyses) {20b}

No prespecified subgroup analyses are planned. However, sensitivity analyses will be performed to assess the uncertainty surrounding cost estimates and to identify the key assumptions that drive those costs, by adjusting various input parameters.

### Methods in analysis to handle protocol non-adherence and any statistical methods to handle missing data {20c}

Every effort will be made to collect full follow-up data for all participants, so it is expected that missing data will be minimal. Procedures to account for protocol non-adherence and missing, unused, or spurious data will be detailed in separate Statistical Analysis Plans, which will be developed and finalised before any analyses are performed.

### Plans to give access to the full protocol, participant-level data and statistical code {31c}

For the data analysis, only the statistician will have full access to the whole dataset and the code to manage the data (cleaning and analysis) while the trial is ongoing. At the end of the studies, site-specific data will be made available to the research teams at the trial sites. In addition, reasonable requests for the protocol and/or trial data from academic parties will be considered by the corresponding author of this manuscript, and data may be made available in line with the data-sharing policies of FIND.

## Oversight and monitoring

### Composition of the coordinating centre and trial steering committee {5d}

As a pragmatic trial with central data management performed by the sponsor (FIND), no coordinating centres or trial steering committees will be required on site.

### Composition of the data monitoring committee, its role and reporting structure {21a}

Ethical review committees in South Africa and Kenya determined that a Data Monitoring Committee was not required, as this trial involves medical devices that already have stringent regulatory approval.

### Adverse event reporting and harms {22}

As outlined in the ‘Outcomes {12}’ section, safety in the trial will be assessed by the recording of AEs and SAEs occurring during the trial periods (from consent up to the final follow-up visit), as defined by CTCAE criteria. All SAEs will be recorded and reported to the trial sponsor (FIND) or their designee within 24 h, and to the IRB/IEC within the required timeframe per IRB/IEC protocols. FIND will comply with all country-specific regulatory requirements relating to safety reporting, to the relevant regulatory authority, and Investigators. All SAEs will be followed until resolution, stabilisation, the event is otherwise explained, or the participant is lost to follow-up, and the Investigator will report any further information regarding the SAE to FIND within 24 h of it being available.

Medical device incidents (limited to the CGM) will also be reported to FIND within 24 h of the Investigators determining that the event meets the Kenya Pharmacy and Poisons Board or South African Health Products Regulatory Authority definition of a medical device incident. All medical device incidents involving an SAE will be followed-up and reported in the same manner as an SAE.

FIND has established an internal Medical Review Board with the overarching role to collaborate actively with the Investigators to protect the safety of trial participants. Monitoring visits will be conducted to assess the implementation of trial procedures, coordination of staff and resources, and data source verification.

The trial sponsor (FIND) will be responsible for the monitoring of recruitment, trial-related training, and central data management, including risk-based monitoring, regular quality control checks, data cleaning, and protocol compliance. The site research teams will be responsible for conducting all trial-related activities at their recruitment sites, and for entering their data into the central EDC system. The trial Investigators will be responsible for verifying that these data entries are accurate and correct, and that the data entered in the EDC system are consistent with the source documents (or the discrepancies adequately explained). Each trial site will be responsible for performing regular quality control checks on the data they generate.

### Frequency and plans for auditing trial conduct {23}

Group meetings will be held weekly to provide updates on the protocol implementation at various sites and to discuss any challenges encountered during this process. Each site will also conduct weekly meetings with selected members of our team to ensure ongoing communication and problem-solving. The Project Management Group will convene quarterly with the whole team at FIND, to review the trial's progress, overall conduct, and integrity. Every three months, a Collaborative Case Study Forum will be organized with all team members from all countries to discuss challenges faced across all sites, facilitating a shared learning environment. The Ethics Committees will meet annually to review the study's progress report, ensuring that ethical standards are maintained throughout the trial. While an external audit is not planned for this trial, FIND will perform risk-based monitoring and scheduled monitoring visits at all sites. This approach will ensure oversight and adherence to trial standards without an external audit.

### Plans for communicating important protocol amendments to relevant parties (e.g. trial participants, ethical committees) {25}

Any amendments to the trial protocols or to other relevant documents (e.g. informed consent forms) must be submitted to an IRB/ERC by the Investigator. Once approved by the sponsor (FIND), amended trial protocols will then be re-signed by the Investigators.

### Dissemination plans {31a}

The results will be communicated to participants, healthcare providers and the scientific community. This study was co-created from a multi-stakeholder workshop including civil societies, Ministries of Health, healthcare professionals, private funders and stakeholders in both countries. During the implementation phase, we will host workshops with the stakeholder groups in each country. Once the study is completed, the results will be shared/presented at local congresses, e.g., the Society for Endocrinology, Metabolism and Diabetes of South Africa (SEMDSA), the Kenya Diabetes Study Group (KDSG) and the East Africa Diabetes Study Group (EADSG) congresses, and local civil society interventions such as South Africa Diabetes Advocacy and Young Unsweet podcasts. Additionally, national dissemination meetings are planned to take place in both countries, including national stakeholders, community representatives, and local staff members. The results will also be disseminated to the scientific community via peer-reviewed publications and presentations at national and international conferences. Manuscripts will be published in open access journals. Dissemination to participants will be via a lay summary with key study findings, to make it understandable and available to all participants; this will be distributed at study sites. Due to previous experiences with restrictions from ethics committees on publishing data in public repositories without explicit participant consent, we will consider the use of a public data repository with the local ethical committees that approved the protocols. Nonetheless, all anonymised data will be available from the corresponding author upon reasonable request. Lastly, in response to the evolving nature of our trial, we commit to regularly updating the online trial registers when there are substantial changes to be reported.

## Discussion

As described earlier in the ‘Background and rationale {6a}’ section, there exists a substantial evidence gap on the use of CGM in people with T1D in LMICs. Results from this trial will help to fill this evidence gap, by providing robust data on the impact of CGM device use in people with T1D in South Africa and Kenya and establishing how CGM compares with standard of care with regard to glycaemic control and HRQoL outcomes. It is also hoped that the empiric evidence provided by this trial will help inform policy decisions, decision-making and treatment practices in these regions.

## Trial status

The current versions of the protocols are version 4.0 (4 September 2023; Kenya), version 1.1 (16 July 2023; Pretoria, South Africa) and version 1.2 (28 September 2023; Cape Town, South Africa). In South Africa, Steve Biko Academic Hospital in Pretoria started recruitment in September 2023. In South Africa, the diabetes clinic at Groote Schuur Hospital and the paediatric diabetes clinic at Red Cross War Memorial Children’s Hospital in Cape Town started recruitment in January 2024. The study in Kenya will start recruitment in June 2024. Study completion is expected in December 2025.

## Data Availability

The only individual with access to the full dataset during the conduction of the trial will be the statistician at FIND. At the end of the trial, site-specific data will be made available to the research teams at the trial sites. Reasonable requests for the trial data from academic parties will be considered by the corresponding author, and data may be made available in line with the data-sharing policies of FIND.

## Abbreviations

%CV: Coefficient of variation
AE: Adverse event
AMREF: The African Medical and Research Foundation
ANOVA: Analysis of variance
CE: European Comission, from “Conformité Européenne”
CGM: Continuous glucose monitoring
CI: Confidence interval
CRF: Case report form
CTCAE: Common Terminology Criteria for Adverse Events
DDS: Diabetes distress score
EDC: Electronic data capture
ESRC: Economic and Social Research Council
FDA: US Food and Drug Administration
GMSS-T1D: Glucose Monitoring Satisfaction Survey for type 1 diabetes
HbA1c: Glycated haemoglobin
HRQoL: Health-related quality of life
ICER: Incremental cost-effectiveness ratio
IEC: Independent Ethics Committee
IRB: Institutional Review Board
LMIC: low- and middle income countries
NACOSTI: National Commission for Science, Technology and Innovation
QALY: Quality adjusted life year
SAE: Serious adverse event
T1D: Type 1 Diabetes
